# Structural Colors on Al Surface via Capped Cu-Si_3_N_4_ Bilayer Structure

**DOI:** 10.3390/mi14020471

**Published:** 2023-02-18

**Authors:** M. A. Rahman, Dongkyu Kim, Deepshikha Arora, Joo-Youl Huh, Ji Young Byun

**Affiliations:** 1 Extreme Materials Research Center, Korea Institute of Science & Technology, 5, Hwarang-ro 14-gil, Seongbuk-gu, Seoul 02792, Republic of Korea; 2 Department of Materials Science and Engineering, Korea University, 145, Anam-ro, Seongbuk-gu, Seoul 02841, Republic of Korea; 3 Engineering Product Development, Singapore University of Technology and Design, 8 Somapah Road, Singapore 487372, Singapore

**Keywords:** reflection colors, metal-insulator-metal structure, Cu thin film, capping layer

## Abstract

Tunable structural colors have a multitude of applications in the beautification of mobile devices, in the decoration of artwork, and in the creation of color filters. In this paper, we describe a Metal-Insulator-Metal (MIM) design that can be used to systematically tune structural colors by altering the thickness of the top metal and intermediate insulator. Cu and Si_3_N_4_ were selected as the top metal and intermediate insulator layers, respectively, and various reflection colors were printed on Al. To protect the Cu surface from scratchiness and oxidation, a number of capping layers, including SiO_2_, LPSQ, PMMA, and the commercially available clear coat ProtectaClear, were applied. In addition to their ability to protect Cu from a humid environment without deteriorating color quality, ProtectaClear and LPSQ coatings have minimal angle dependency. Furthermore, a bilayer of PMMA/SiO_2_ can protect the Cu surface from the effects of humidity. In addition, the PMMA/SiO_2_ and ProtectaClear/SiO_2_ bilayers can also protect against corrosion on the Cu surface. The colors can be tuned by controlling the thickness of either the metal layer or intermediate insulator layer, and vivid structural colors including brown, dark orange, blue, violet, magenta, cyan, green-yellow, and yellow colors can be printed. The measured dielectric functions of Cu thin films do not provide any evidence of the plasmonic effect, and therefore, it is expected that the obtained colors are attributed to thin-film interference.

## 1. Introduction

Structural colors became much more attractive to researchers as it appears more aesthetic than other traditional coloration like pigment or dye-based colors and also have a long-lasting capability in color quality [1]. Therefore, the structural coloration of metals can be used in the beautification of mobile devices, art decoration, and color filters [2,3,4]. Several methods have been reported to date to prepare structural colors such as bio-inspired photonic structures [5,6,7], diffraction gratings [8], plasmonic, and dielectric-based structures [9,10,11,12,13,14,15,16]. But these techniques require complicated procedures, e.g., nanoscale lithography. Recently, structural color preparation by asymmetric Fabry−Perot type structures is also proposed [17,18,19,20,21,22,23,24,25,26]. The advantage of these F-P cavity structures is it does not require nano-patterning to fabricate colors. Additionally, colors can be tuned very easily by the thickness of the intermediate layer.

The present research work is further work of our recent report on structural color preparation by a non-expensive Cu/Si_3_N_4_/Cu structure [26]. In a previous report, it was shown that a range of highly saturated colors could be generated by Cu/Si_3_N_4_/Cu structure with the introduction of capping layers on top for protecting the structure from corrosion. In the present study, structural colors were realized on the widely used Al substrate. The Cu/Si_3_N_4_ bilayer was coated on an Al back reflector and colors were tuned by tuning the thickness of the top Cu and intermediate Si_3_N_4_ layer. Various colors—that is, brown, dark orange, blue/magenta, cyan, green-yellow, and yellow color—can be printed on the Al surface by tuning the Si_3_N_4_ intermediate layer. The color can also be slightly tuned by tuning Cu thickness. To protect the colors from harsh environments SiO_2_, commercial ProtectaClear (PC), Polymethyl methacrylate (PMMA), and ladder-like Polysilsesquioxanes (LPSQ) of several thicknesses were introduced. The color quality does not degrade after coating these capping layers on the MIM structures and colors can also be protected from the hard corrosive environment. The analytical calculations were carried out to calculate reflectance and pseudo colors using the refractive indices of measured values of Cu thin films. The reflectance spectra of the MIM structures were calculated by OpenFilters software, which adapts the characteristic matrix approach. The calculated reflectance spectra matched reasonably with experimental data. The measured dielectric functions of Cu thin films do not exhibit any clear evidence of plasmonic effect in the visible range of wavelength and therefore, the interference effect was considered solely responsible for printing these structural colors.

## 2. Materials and Methods

To fabricate a Cu/Si_3_N_4_/Al trilayer structure on a Si substrate, optically thick Al (200 nm) thin film was deposited by electron beam evaporation technique (EBX-1000, ULVAC, Japan) and onto a polished (1 1 1) Si wafer, which was used as a bottom metal surface of the MIM structure. Prior to the deposition, the chamber was evacuated until it reaches a pressure of 3 × 10^−7^ Torr. Afterward, the dielectric Si_3_N_4_ layer (30–130 nm) was deposited using a plasma-enhanced chemical vapor deposition (PECVD) system (PlasmaPro 800Plus, Oxford Instruments Plasma Technology (OIPT), Bristol, UK). For the Si_3_N_4_ deposition, a mixture of SiH_4_, NH_3_, and N_2_ was used, and the substrate temperature was maintained at 250 °C. Afterward, Cu thin films of various thicknesses (12, 15, 18, 25, 30, and 35 nm) were deposited on top of the Si_3_N_4_ layer by a magnetron sputtering method and the MIM structures were prepared. Prior to the deposition of the Cu top layer, the chamber of magnetron sputtering was evacuated to 1.5×10^−6^ Torr. The film thicknesses of Cu were varied by deposition time and the deposition rate was controlled at 0.20 nm/s. The Cu film was deposited at room temperature under a working pressure and sputter power of 2 mTorr and 40 W, respectively. The refractive indices of the Cu films were measured by the ellipsometry method. Au thin film was also deposited by DC magnetron sputtering under a base pressure of 2.0 × 10^−6^ Torr while DC power of 20 W was used to deposit 10 nm film on the Si_3_N_4_/Al bilayer. The SiO_2_ insulating layers were deposited on top of MIM structures. The SiO_2_ thin films were also deposited via the PECVD technique (Oxford Plasma Pro 800 plus). The dip-coating method was used to coat commercially available ProtectaClear and PMMA coats while LPSQ was coated by drop casting method. PC was purchased from Everbrite, Inc., Rancho Cordova, CA, USA, and PMMA solutions were purchased from Kayaku Advanced Materials (Japan). A custom-made humidity chamber was prepared for the humidity experiment. A plastic container was taken as a humidity chamber where half of it was filled with water. Later, all MIM samples were kept as they float in the chamber, and the plastic box was firmly closed. Finally, the humidity chamber was kept inside a drying oven at 60 °C for 24 h. A salt spray test was carried out to study the corrosion of the MIM structure according to the standard ASTM B-117. The ASTM stands for American Society for Testing and Materials and the organization develops and publishes the technical standards for a variety of materials. ASTM B-117 is one of the certified methods from ASTM internationals for knowing corrosion resistance information of metals or coated metals. The corrosion test was performed in an Erichsen Corrocompact 617 apparatus (Erichsen GmbH & Co. KG, Hemer, Germany) with. All the specimens were kept in the enclosed chamber and exposed to a 5% NaCl solution. The temperature during the salt spray experiment was kept at 35 °C and the test period was 24 h.

The cross-sectional TEM specimens were prepared by the focused ion beam (FIB) milling method and samples were characterized via a transmission electron microscope (FEI Talos F200X S/TEM, Thermo Fisher Scientific (TMO), Waltham, MA, USA). The plan-view microstructures were characterized using a field-emission scanning electron microscope (FEI Inspect F50, FEI, Hillsboro, OR, USA). The reflectance spectra of the fabricated samples were measured using a spectrophotometer with a xenon white source of beam diameter 4 mm (CM 3600A, Konica Minolta, Inc., Tokyo, Japan), for which the detector and the incident light beam were positioned at 8° from the surface normal. To calculate the reflectance spectra, the OpenFilters software [27] was used with inputs of the refractive indices and thicknesses of the individual layers in the multilayer structure. The Spectramagic NX software was used to extract the pseudo colors of the fabricated samples obtained from their reflectance spectra [28]. The Spectramagic NX software was also used to obtain the simulated colors with the inputs of the calculated reflectance. For capturing color images of all the specimens, a Canon 500D camera was used, and images were captured under a fluorescent lamp.

## 3. Results

### 3.1. Printing Structural Colors by Cu-Based MIM Structures

**Figure 1:**
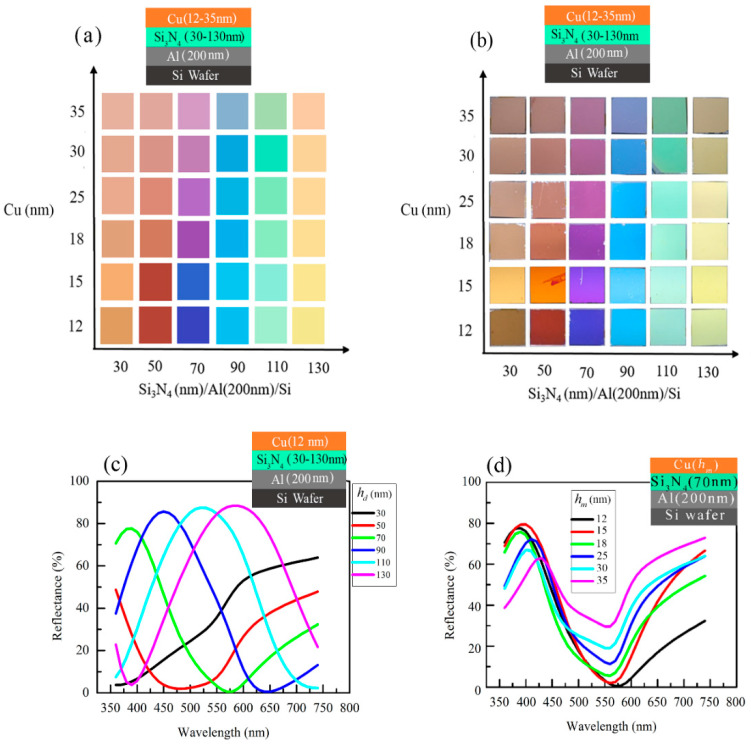
(**a**) The measured structural color of the Cu($h_{m})$/Si_3_N_4_($h_{d}$)/Al (200 nm)/Si structures with varying $h_{m}$ = 12–35 nm and$\;h_{d}=$ 30–130 nm in the step of 20 nm. (**b**)The camera images of the same. The dependencies of the reflectance spectra with varying (**c**) The dependencies of the reflectance spectra with varying $h_{d}\;$in Cu (12 nm)/Si_3_N_4_($h_{d}$)/Al (200 nm)/Si structures and (**d**) $h_{m}\;$in Cu ($h_{m})$/Si_3_N_4_ (70 nm)/Al (200 nm)/Si structures.

The measured colors and captured images of the Cu(*h_m_*)/Si_3_N_4_(*h_d_*)/Al (200 nm) structures with varying thicknesses of *h_m_* =12–35 nm and *h_d_* = 30–130 nm in steps of 20 nm are illustrated in Figure 1a,b. In this case, *h_m_* was increased from 12 to 15, 18, 25, 30, and 35 nm. By varying the thickness of Cu and Si_3_N_4_ layers, a variety of colors, including brown, dark orange, blue, violet, magenta, cyan, green-yellow, and yellow, can be printed. A slight variation is noticed between the measured colors and the images captured by a digital camera because, during the capturing images, brightness might be slightly varied if compared to the measured color. On the other hand, in the case of measured color, the specimen is kept in a controlled way so that we can get the actual color of the specimen. Therefore, we should consider the measured colors as a more accurate measure. Reflectance spectra with variation in Cu (12 nm)/Si_3_N_4_(*h_d_*)/Al (200 nm)/Si structures are presented in Figure 1c. As the wavelength increases, the resonance absorption at the reflectance dip redshifts. The Cu/Si_3_N_4_(*h_d_*)/Al structure produces a change in color due to the shift in the reflectance dip. This can be understood from F-P cavity resonances. In this case, the reflectance dips shift towards red wavelengths with an enhancement for a fixed *h_m_*. The resonance condition in the F-P cavity of a MIM structure can be expressed as [18]

(1) $$\left(\frac{4\pi }{\lambda_{res}}\right)n_{d}h_{d}+\phi_{b}+\phi_{t}=2m\pi$$

where $\phi_{b}$ $\mathrm{and}\;\phi_{t}$ are the phase shifts upon reflection at the interfaces of the dielectric layer with the bottom and top metal thin film layers, respectively, $n_{d}$ and $h_{d}$ are the reflective index and thickness of the dielectric layer, and $\lambda_{res}$ is the resonance wavelength, respectively, and m is an integer that represents the order of the cavity mode. In Equation (1), the subscripts d, b, and t represent dielectric, bottom, and top respectively. As the reflection phase shifts $\phi_{b}$ and $\phi_{t}$ both depend on the refractive indices and thicknesses of the respective metal thin film layers, Equation (1) expresses a linear increase $\lambda_{res}$ with $h_{d}$ can be observed for a fixed *h_m_* and the bottom Al layer thickness (200 nm), which eventually leads to a redshift of the reflection dip as $h_{d}$ are increased, and this can be observed in Figure 1. The dependencies of the reflectance spectra with varying $h_{m}$ in Cu ($h_{m})$/Si_3_N_4_ (70 nm)/Al (200 nm)/Si structures are presented in Figure 1d. The absorption is noticed at 550–570 nm wavelength depending on the *h_m_* and the absorption decreased as *h_m_* increased. A blueish color is realized due to comparatively higher R% values being noticed in the blue wavelength region of wavelength. When *h_m_* increased, the R% enhanced in the longer visible wavelength, and the absorption region slightly shifted to the blue wavelength region. This phenomenon turned the color to blue-magenta and pink-magenta as *h_m_* were increased. As *h_m_* increases, the absorption at the reflectance dip decreases linearly.

**Figure 2:**
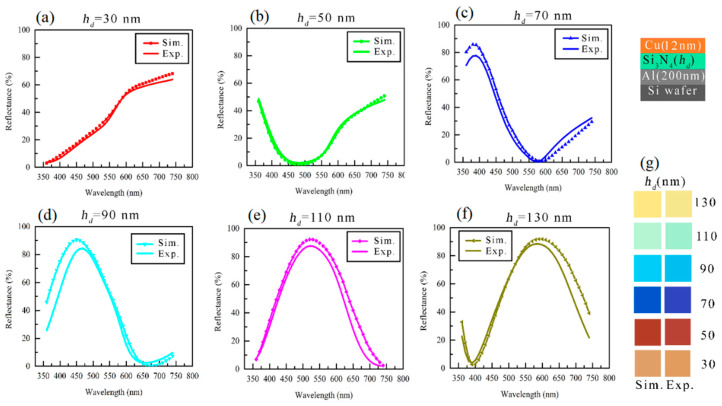
The simulated vs experimental reflectance spectra of Cu (12 nm)/Si_3_N_4_(*h_d_*)/Al (200 nm) structure with increasing *h_d_*: (**a**) *h_d_* = 30 nm, (**b**) *h_d_* = 50 nm, (**c**) *h_d_* = 70 nm, (**d**) *h_d_* = 90 nm, (**e**) *h_d_* = 110 nm and (**f**) *h_d_* = 130 nm. (**g**) Simulated versus experimental colors of the same structure of (**a**–**f**).

**Figure 3:**
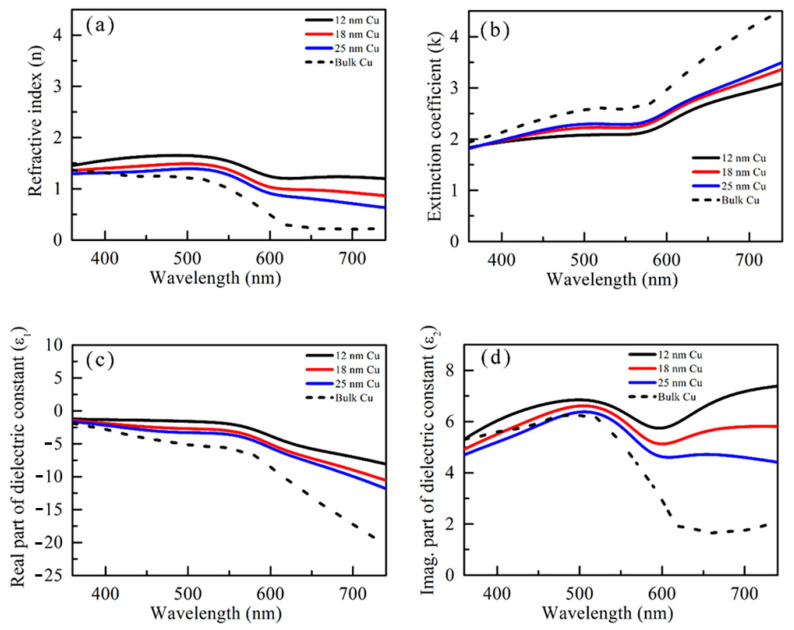
(**a**) The refractive indices (n), (**b**) the extinction coefficients (k), (**c**) the real ($\varepsilon_{1}),$ and (**d**) imaginary ($\varepsilon_{1})$ parts of the dielectric constants of 12 nm, 18 nm, and 25 nm Cu film measured by ellipsometry method. These values are also compared with refractive indices and dielectric functions of bulk Cu.

Simulations have been carried out to calculate the reflectance and the colors for the Cu (12 nm)/Si_3_N_4_ (*h_d_*)/Al structure, and these are plotted with a measured counterpart in Figure 2a–f and Figure 2g, respectively. The measured refractive indices of Cu that were used to simulate reflectance spectra and colors are shown in Figure 3. To simulate the reflectance spectra, previously reported refractive indices of bulk Al and of the Si_3_N_4_ were used [17,29]. Here, the thicknesses of each layer for the experimental MIM structure were measured by TEM imaging and used for simulation. The simulation data supports reasonably well with the experimental counterparts.

Thickness-dependent dielectric functions of Cu thin films along with their bulk counterparts [30] are plotted in Figure 3. The $\varepsilon_{1}$ values exhibit negative values in the whole range of wavelength for 12–25 nm thick Cu films. A negative $\varepsilon_{1}$ value suggests that light cannot deeply penetrate as it has a large k value. The $\varepsilon_{1}$ spectra for the 12 nm thick Cu film exhibit broad peak-like nature at ~720 nm, and this extended over the visible range. As Cu thicknesses are increased, the $\varepsilon_{2}$ value reduces in longer wavelengths as it is observed in bulk Cu. The Cu thin film exhibits a surface plasmonic band at 770–850 nm, depending on the size of the nanoparticle [31]. The extended peak-like nature shown by the 12 nm Cu film might be due to the plasmonic effect, but according to the dielectric spectra, the SPR peak exists at a longer wavelength, possibly at >740 nm. Therefore, Cu films do not provide any clear evidence of having a plasmonic effect in the visible range. Thus, colors that appear from the MIM structure are realized due to the sole contribution of the interference effect.

### 3.2. Protecting Cu Surface by Capping Layers

**Figure 4:**
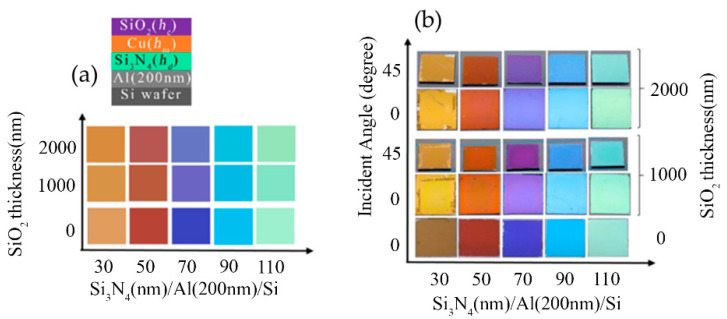
(**a**) The measured pseudo colors and (**b**) the camera images of the SiO_2_ (1000–2000 nm)/Cu (*h_m_*)/Si_3_N_4_(*h_d_*)_/_Al structure.

**Figure 5:**
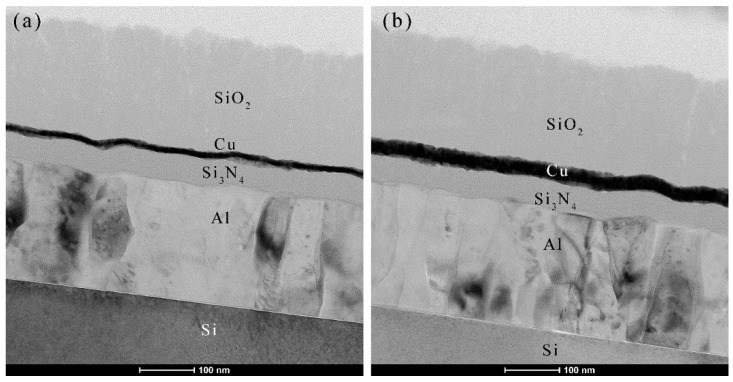
The Cross-sectional TEM images of (**a**) SiO_2_ (200 nm)/Cu (12 nm)/Si_3_N_4_ (50 nm)/Al (200 nm)/Si and (**b**) SiO_2_ (200 nm)/Cu (25 nm)/Si_3_N_4_ (50 nm)/Al (200 nm)/Si structures.

The SiO_2_ thin film was chosen as one of the capping layers on Cu(*h_m_*)/Si_3_N_4_(*h_c_*)/Al MIM measured pseudo color and camera images were shown in Figure 4a,b, respectively. Herein, thicknesses of SiO_2_ (*h_c_*), Cu (*h_m_*), and layer Si_3_N_4_ (*h_d_*) were chosen as 1–2 μm, 12 nm, and 30–130 nm, respectively. For the SiO_2_ layer with a thickness of 1–2 μm, excellent pseudo colors were obtained, which almost kept the original color of the uncapped Cu/Si_3_N_4_/Al. The view-angle dependency of the thick SiO_2_-coated MIM structures was studied, and it exhibits minimal angle dependency behavior (Figure 4b). The cross-sectional TEM images of SiO_2_ (200 nm)/Cu (12 nm)/Si_3_N_4_ (50 nm)/Al/Si and SiO_2_ (200 nm)/Cu (25 nm)/Si_3_N_4_ (50 nm)/Al/Si structures are shown in Figure 5 and it is observed that both Cu thin films exhibit continuous growth mode.

**Figure 6:**
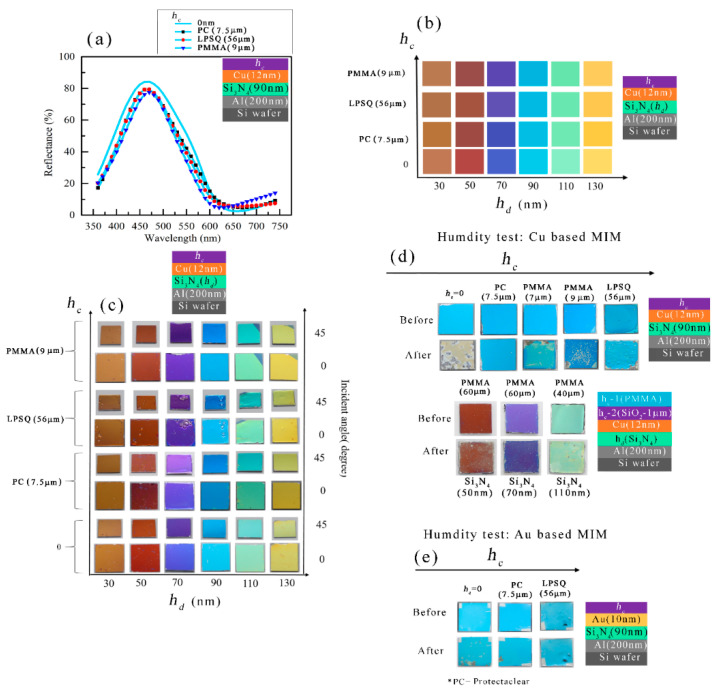
The reflectance spectra and structural colors of *h_c_*/Cu (12 nm)/Si_3_N_4_ (90 nm)/Al (200 nm/Si structures where three different passivation layers were used as *h_c_*: Protectaclear coat (PC), LPSQ, and PMMA. (**a)** The reflectance spectra of *h_c_*/Cu (12 nm)/Si_3_N_4_ (90 nm)/Al (200 nm/Si MIM structures with three different passivation layers compared with uncoated structure. (**b**)The measured colors of the *h_c_*/Cu (12 nm)/Si_3_N_4_(*h_d_*)/Al (200 nm/Si MIM structures. (**c**) The color images were captured by a digital camera at 0 degrees and a 45-degree incident angle for the same structure of (**b**). The captured images of the before and after humidity test of the (**d**) *h_c_*/Cu (12 nm)/Si_3_N_4_ (90 nm)/Al (200 nm)/Si and *h_c_* -1/*h_c_* -2/Cu (12 nm)/Si_3_N_4_ (90 nm)/Al (200 nm)/Si structures and of the (**e**) *h_c_*/Au (10 nm)/Si_3_N_4_ (90 nm)/Al (200 nm/Si MIM structures.

A few other transparent polymer layers, such as PMMA, LPSQ, and ProtectaClear (PC), were also chosen as capping layers of the MIM structure, and a comparative study is shown in Figure 6. Among the capping layers that we use, SiO_2_ is known as hydrophilic and other polymers are hydrophobic. Figure 6a shows the reflectance spectra of *h_c_*/Cu (15 nm)/Si_3_N_4_ (90 nm)/Al (200 nm)/Si MIM structures where three different passivation layers were used as *h_c_*: 7.5 μm thick PC, 56 µm thick LPSQ, and 9 µm thick PMMA. As can be observed, the pattern of the reflectance spectra was unchanged while coated in these thick protective layers and, therefore, original colors could be realized with the capping layers. Figure 6b,c shows the measured pseudo colors and camera images of the *h_c_*/Cu (15 nm)/Si_3_N_4_(*h_d_*)/Al (200 nm)/Si structure which state that color was not altered after these coating of capping layers. The measured pseudo colors show that the brown, orange, blue, cyan, green-yellow, and yellow colors could be printed without a capping layer. The PC-coated MIM structures could keep the exact colors obtained by uncapped (*h_c_*=0) MIM structures. The PMMA and LPSQ also show good candidacy to keep the original colors but a slight shift in colors from blue to violet is noticed in the case of *h_c_*/Cu (15 nm)/Si_3_N_4_ (70 nm)/Al (200 nm)/Si structure. Figure 6c shows the camera images taken for the PC, LPSQ, and PMMA-coated MIM structures, and photos were captured both vertically (incident angle:0-degrees) and angularly (incident angle: ~45 degrees). The view-angle-dependency in colors are shown for the *h_c_*/Cu (15 nm)/Si_3_N_4_(*h_d_*)/Al (200 nm)/Si structures, and view-angle-dependency is found to be negligible while coated PC, LPSQ, and PMMA. The *h_c_*/Cu (15 nm)/Si_3_N_4_ (70 nm)/Al (200 nm)/Si structure shows a blue to pink-violet shift in colors. This happened due to the case that uncapped Cu (15 nm)/Si_3_N_4_ (70 nm)/Al (200 nm)/Si shows slight angle dependency: blue (incident angle:0-degrees) and pink-violet (incident angle: 45 degrees).

### 3.3. Reliability Test of MIM Structures 

A humidity test was carried out to see whether these capping layers can protect the colors in harsh environments. Figure 6d shows before and after the humidity test for the PC coated (7.5 µm), PMMA (7 and 9 µm), and LPSQ (56 µm) coated Cu-based MIM structures. As seen in Figure 6d, a 7.5 µm thick PC layer is sufficient to protect Cu-based MIM structure from a humid environment. A 56 µm thick LPSQ-coated layer could also provide similar protection. 7–9 µm thick PMMA layer could not protect the Cu surface from a moist environment where an uncapped Cu-based MIM structure can be easily oxidized, and color was removed from the surface while kept in a humid environment. A combination of PMMA and SiO_2_ layers were used as the capping layers to protect the Cu surface from the humid environment and Cu (12 nm)/*h_d_* (Si_3_N_4_)/Al structures were studied. A SiO_2_ (1 µm) layer is considered as 1st layer of capping and PMMA (40–60 µm) layers were used as the top layer. The 60 µm thick PMMA layer was found to be protective enough from the humid environment and the colors of the three samples i.e., *h_d_* =50 nm, 70 nm, and 110 nm remained intact. The vertically taken images of the PMMA/SiO_2_ bi-layer coated MIM structure were shown in Figure 6d. The colors remained view-angle independent after this PMMA/SiO_2_ bi-layer coating and a similar phenomenon was found after the humidity test.

The capping layers were also coated on Au-based MIM structures (*h_c_*/Au (10 nm)/Si_3_N_4_ (90 nm)/Al (200 nm) and the effect of these capping layers in a humid environment was studied (Figure 6e). After the humidity test, the uncapped Au-based MIM structure exhibited large cracks, while the MIM structures capped by PC (7.5 µm) and LPSQ (56 µm), gave strong protection in a harsh environment. As the Au surface is scratch-sensitive, therefore, PC and LPSQ layers can be excellent candidates for protecting Au-based MIM structures from scratchiness.

**Figure 7:**
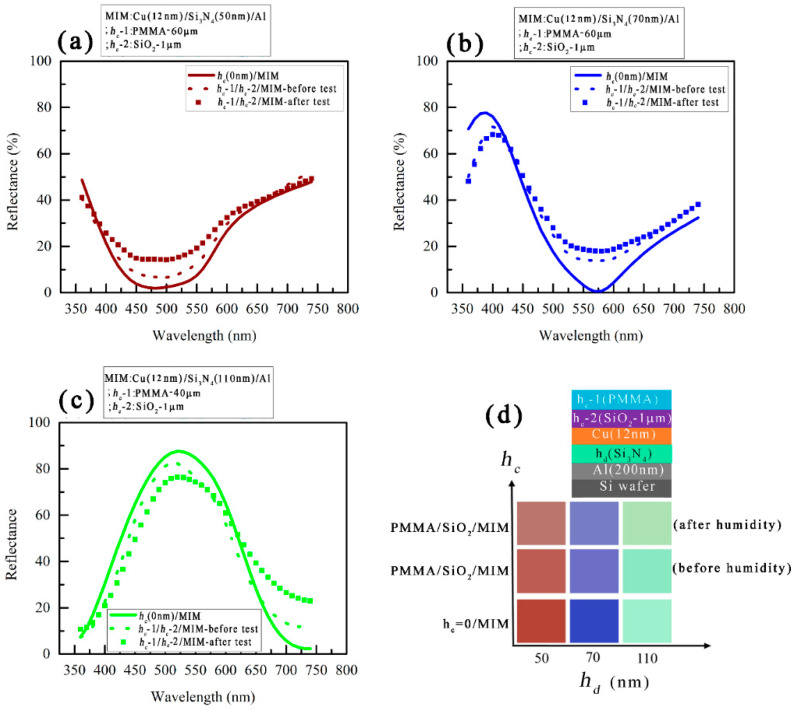
Reflectance spectra of uncoated and PMMA/SiO_2_ coated MIM structure. The reflectance spectra after the humidity test were presented as well for comparison; (**a**) PMMA (0–60 µm)/SiO_2_ (1 µm)/Cu (12 nm)/Si_3_N_4_ (50 nm)/Al (200 nm)/Si, (**b**) PMMA (0–60 µm)/SiO_2_ (1 µm)/Cu (12 nm)/Si_3_N_4_ (70 nm)/Al (200 nm)/Si, (**c**) PMMA (0–40 µm)/SiO_2_ (1 µm)/Cu (12 nm)/Si_3_N_4_ (110 nm)/Al (200 nm)/Si structure. (**d**) Measured colors of the uncoated and PMMA/SiO_2_ coated MIM structure.

The reflectance spectra and measured colors of the same structure were presented in Figure 7. The reflectance spectra before and after the bi-layer coating of the capping layers (Figure 7a–c) lie close to each other. The reflectance spectra after the humidity test also show a similar trend and therefore, measured colors (Figure 7d) in these three cases i.e., uncoated MIM structure, after the PMMA/SiO_2_ bi-layer coating, and after the humidity tests, remained almost the same.

**Figure 8:**
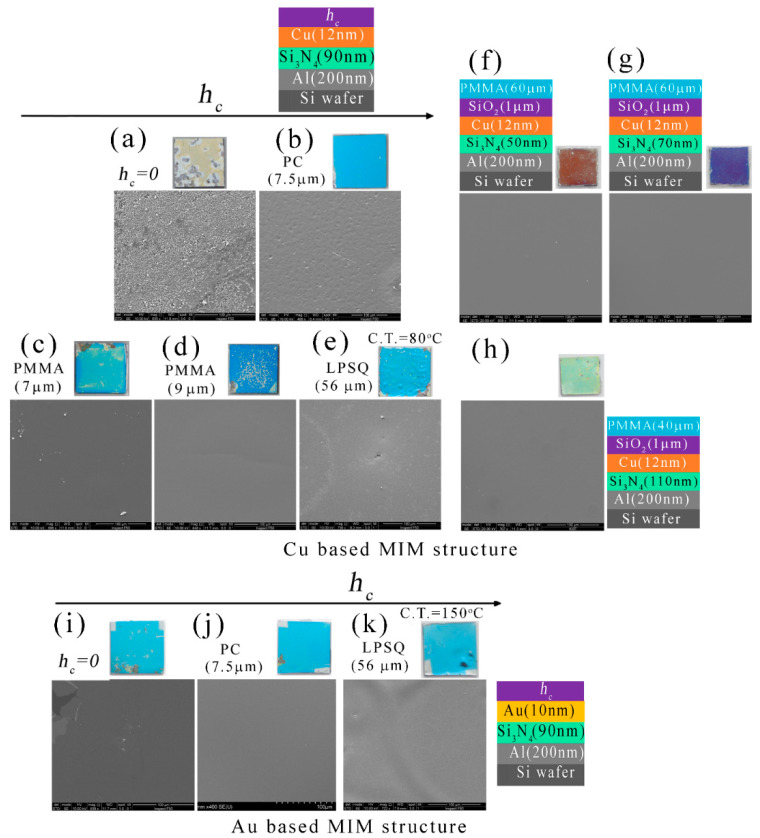
The scanning electron microscopy (SEM) images of the humidity test specimens: (**a**–**h**) Cu-based MIM structures; (**i**–**k**) Au-based MIM structures. The scale bar in all images is 100 μm.

Figure 8 shows the plan-view SEM images of the bare Cu/Si_3_N_4_/Al structures after the humidity test, and these were compared with capped MIM structures. Figure 8a SEM represents the bare Cu/Si_3_N_4_/Al structure where macrostructure from the camera images shows large cracks and voids, and these are clearly noticed in the SEM microstructure. The surface was rough and rusty as it was oxidized in a humid environment. A 7.5 µm thick PC coating can give strong protection from the humid environment as the camera images of the specimen show the color was intact and no crack was observed. The SEM image of the specimen (Figure 8b) shows that it has small pores after the humidity test. Figure 8c,d show the SEM images of 7–9 µm thick PMMA coated Cu/Si_3_N_4_/Al structure. Even though macrostructures observed from the camera images exhibited large cracks and degrade colors, these cracks were not clearly visible in SEM images. LPSQ-coated Cu/Si_3_N_4_/Al structure exhibits a large number of cracks, and these are visible to the naked eye (camera images of Figure 8e). The LPSQ-coated specimen was cured at 80 °C and could not give good protection under humid conditions. Figure 8e presents the SEM microstructure of the LPSQ-coated Cu/Si_3_N_4_/Al structure and few cracks were noticed in the SEM images. The SEM images of the PMMA/SiO_2_ bi-layer coated Cu-based MIM structure after the humid test were presented in Figure 8f–h and all three samples exhibited very smooth surfaces after the test. This signifies that a combination of polymer and CVD-coated transparent bilayer can protect sufficiently from the humid environment.

The SEM images of the capped and uncapped Au (10 nm)/Si_3_N_4_ (90 nm)/Al structure after the humidity test, are shown in Figure 8i–k. The SEM image of the uncapped Au-based MIM structure is shown in Figure 8i. The color was unchanged after the humidity test in the cases of the uncapped Au/Si_3_N_4_/Al structure but there are some voids or cracks visible in the macrostructure. The SEM microstructure of the specimen shows a large crack in the top left corner of the image, and it is representative of one of the cracks visible by the naked eye. Figure 8j represents a 7.5 µm thick PC-coated Au/Si_3_N_4_/Al structure and it shows a very good smooth surface from the SEM images. The 56 µm thick LPSQ layer coated Au/Si_3_N_4_/Al structure (Figure 8k) provided a smooth surface that was visible in both the SEM images and in the macrostructure. After the LPSQ coating, the Au/Si_3_N_4_/Al structure was cured at 150 °C in a vacuum with continuous Ar flow.

**Figure 9:**
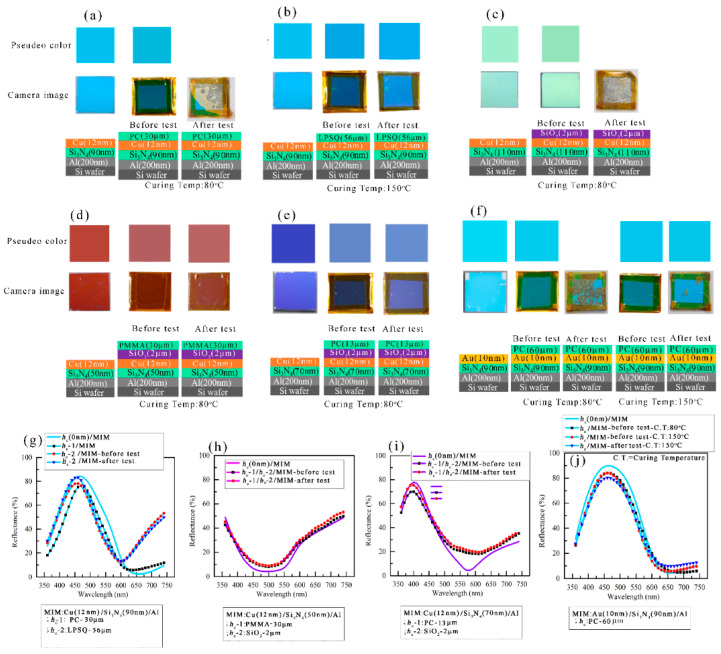
The camera images and pseudo colors of the salt spray test specimens: (**a**–**e**) Cu-based MIM structures; (**f**) Au-based MIM structures. (**g**–**j**) The reflectance spectra of the MIM structures before and after the salt spray test.

Figure 9 represents the pseudo colors and camera images before and after the salt spray test of the capped MIM structures. The cyan color appeared in bare Cu (12 nm)/Si_3_N_4_ (90 nm)/Al structure and after a 30 μm thick PC was coated on the MIM structure, the color remained almost the same. The reflectance spectra of the PC-coated MIM structure almost closely matched before and after the PC coating (Figure 9g). The PC-coated Cu-based MIM structure could not pass the salt spray test as 90% of the surface of the PC-coated MIM structure was corroded after the test which can be seen in Figure 9a. LPSQ coating was used as well to understand how protective this layer can be in a salty environment. In the case of the humidity test of the specimens, it was observed that 80 °C was not the standard curing temperature as it failed to give accurate protection from the humid environment. Therefore, a 56 µm thick LPSQ-coated Cu (12 nm)/Si_3_N_4_ (90 nm)/Al structure was cured at 150 °C in a vacuum furnace with the flow of Ar gas. Figure 9b shows a cyan-colored sample of the same MIM structure as in Figure 9a, where LPSQ was coated as a capping layer. After curing the LPSQ-coated MIM structure, a slight color variation is noticed while the cyan color is slightly varied to a blue-ish cyan color. A slight change in reflectance was also noticed when LPSQ was coated on the MIM structure as compared with the uncoated MIM structure, where reflectance values largely enhanced in longer wavelength (600–740 nm). The reflectance peak position remained almost the same in both uncoated and LPSQ-coated structures and thus bluish color remained. This slight color change in LPSQ coated Cu/Si_3_N_4_/Al structure occurred possibly due to the fact the specimen might be slightly oxidized during the curing process. The color and surface remained intact even after the salt spray test for the case of LPSQ. The reflectance spectra of the uncoated MIM structure are compared with the reflectance spectra obtained from the LPSQ-coated MIM structure before and after the salt spray test (Figure 9g) and it is observed that the reflectance spectra before and after the salt spray test almost remained constant. A green-ish colored Cu (12 nm)/Si_3_N_4_ (110 nm)/Al structure is noticed in Figure 9c to keep the original color when coated with SiO_2_ (2 μm) layer as the capping layer of the MIM structure but failed to keep the color rather surface is completely corroded during salt spray test. An orange-colored Cu (12 nm)/Si_3_N_4_ (50 nm)/Al structure was shown in Figure 9d, where color remained almost the same after PMMA (30 nm)/SiO_2_ (2 µm) bilayer was coated on Cu/Si_3_N_4_/Al structure and color also remained intact even after salt spray test. The reflectance spectra of uncoated Cu (12 nm)/Si_3_N_4_ (50 nm)/Al structure were compared with PMMA/SiO_2_ bilayer-coated MIM structure before and after the salt spray test (Figure 9h). A small variation is noticed, i.e., the reflectance value in the reflectance dip region slightly enhanced as PMMA/SiO_2_ was coated on the MIM structure and therefore, the difference in the color before and after the coating of the capping layer is less significant. Reflectance spectra closely overlapped each other before and after the salt spray test which signifies that color quality was intact after the salt spray test. A bare Cu (12 nm)/Si_3_N_4_(70 nm)/Al structure exhibited violet color when a PC (13 μm)/SiO_2_ (2 μm) bilayer was coated as a passivation layer on the MIM structure (Figure 9e), the color brightness of the specimen was enhanced as the reflectance value in mid to longer wavelength (550–750 nm) region. The reflectance spectra (Figure 9i) remained almost the same before and after the salt spray test which also produced an almost similar color.

The corrosion test was also carried out for the specimens capped by various capping layers on Au (10 nm)/Si_3_N_4_ (90 nm)/Al structure. Figure 9f shows that cyan color can be realized on the Au (10 nm)/Si_3_N_4_ (90 nm)/Al structure and color remained almost constant while coated PC (60 μm)/SiO_2_ (2 μm) bilayer as the capping layer. The capped MIM structure was cured at two temperatures: 80 °C and 150 °C in a drying oven and the color of the specimens were found to be similar in both conditions. The capped MIM structure cured at 80 °C was damaged in the salt spray test while a large amount of Au surface was intact in the case of the specimen which was cured at 150 °C. Figure 9j represents the reflectance spectra of the PC (60 μm) coated Au (10 nm)/Si_3_N_4_ (90 nm)/Al/Si structure cured at 80 °C and 150 °C while these spectra were compared with uncoated Au/Si_3_N_4_/Al structure. The reflectance spectrum of the specimen which passed the salt spray test was also compared with the spectra measured before the test. The reflectance spectra obtained before and after the salt spray test for the specimens cured at 150 °C, matched very closely, thus the color also remained the same.

**Figure 10:**
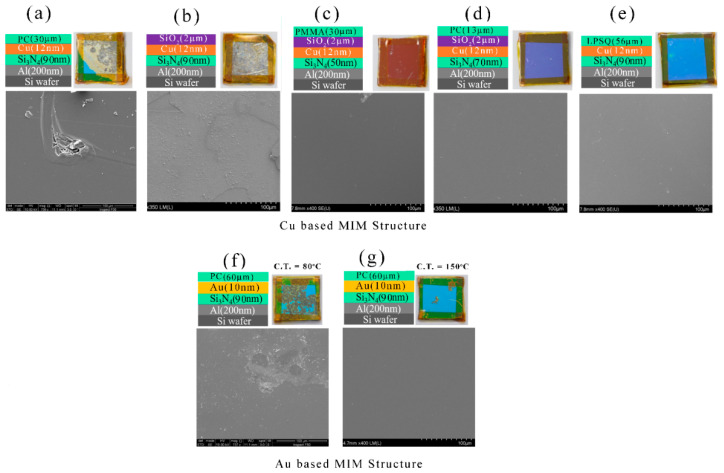
The scanning electron microscopy (SEM) images of the salt spray test specimens: (**a**–**e**) Cu-based MIM structures; (**f**–**g**) Au-based MIM structures. The scale bar in all images is 100 μm.

The plan-view SEM images of the salt spray test specimens are shown in Figure 10. Figure 10a represents the SEM image of the PC-coated specimen after the salt spray test. Large cracks are visible in the SEM microstructures of the corroded specimen. The SiO2-coated Cu/Si_3_N_4_/Al structure was completely corroded and the damaged surface was observed in the SEM image shown in Figure 10b. PMMA/SiO_2_, PC/SiO_2,_ and LPSQ coated Cu/Si_3_N_4_/Al structure passed the salt spray test, and the SEM images of these specimens after the test were shown in Figure 10c–e, respectively. The SEM images show that a very smooth surface can be obtained even after the salt spray test was performed. The plan view SEM images of PC coated Au (10 nm)/Si_3_N_4_ (90 nm)/Al structure cured at 80 °C and 150 °C after the salt spray test, were shown in Figure 10f,g respectively. The specimen cured at 80 °C has large cracks which are noticeable in the camera images and these cracks are found in the SEM images. The specimen cured at 150 °C gives better protection from the salt spray though few cracks were noticed in the macrostructure (camera image), and no voids or cracks were observed in the SEM image rather smooth surface is noticed.

As the capping layers are relatively thick (e.g., order of 10 µm), these can be coated on the structures via vacuum-free coating methods such as the dip coating method. This will be advantageous for the mass production of these color coatings. 

## 4. Conclusions

To summarize, we have proposed a model to prepare reflective structural colors using Cu-based MIM structures on Al back reflectors. By varying the thickness of Cu and Si3N4 layers, several colors can be printed, such as brown, dark orange, blue, violet, magenta, cyan, green-yellows, and yellows. SiO_2_, ProtectaClear, PMMA, and LPSQ were studied as capping layers to protect the Cu surface. SiO_2_ layers 1–2 mm thick can maintain the original color and provide angular independence. In addition to protecting from scratchiness, SiO_2_ can also protect from oxidation in the short term. ProtectaClear, PMMA, and LPSQ present excellent prospects regarding color quality and angle independency. In addition to protecting Au-based MIM structures (60 °C, 24 h), ProtectaClear and LPSQ provide strong protection against moisture (60 °C, 24 h). Even though a single-layer PMMA-coated Cu-based MIM structure could not protect the surface from the humid environment, this problem can be solved by a PMMA/SiO_2_-bilayer coating. A single LPSQ layer can be a good candidate to protect the Cu surface from the hard corrosive environment as it passes the salt spray test (24 h) while ProtectaClear/SiO_2_ and PMMA/SiO2 bilayer play the same role. The ProtectaClear coat is sufficient to protect the Au surface from corrosion. It provides cost-effectiveness, lithography-free design, and color-tuning capability, so it can be used to make real color coatings.

## Data Availability

The data presented in this study are available upon request.
